# Tardiness in the Shedding of the Temporary Teeth

**Published:** 1847-10

**Authors:** 


*Tardiness in the Shedding of the Temporary Teeth.*—It is well known that one or more of the temporary teeth sometimes remain firmly articulated for years, after the time when they are usually removed by the operations of the economy, to give place to their more hardy successors. We have frequently met with individuals, thirty, forty, and even fifty years of age, with two or three of the temporary teeth still remaining in the jaws; but one of the most singular examples of this sort which we recollect to have ever met with, come to our notice a few days since; the subject of which is a lady between twenty-five and thirty years of age. All her temporary teeth were shed and replaced, as she informed us, at about the proper periods, except the left superior lateral incisor and cuspidatus.

These are still securely articulated, and in a perfectly healthy condition, while all of her permanent teeth are more or less affected by caries. The crowns of many are nearly destroyed. There is no indication of the appearance of the permanent lateral incisor, but the point of the crown of the cuspidatus of replacement has pierced the gum above the left central incisor, and the tooth, as is indicated by a ridge on the anterior part of the alveolar border, occupies a horizontal position in the jaw.—*Balt. Ed.*

